# Anxiety of a Dying Man

**Published:** 1884-05

**Authors:** 


### ANXIETY OF A DYING MAN.

Two doctors were disputing by the bedside of a patient. "I tell you the liver is diseased," said one. "Nonsense; nothing of the kind It is the spleen." "Very well; we shall see at the post mortem who is in the right." Great sensation on the part of the patient, whom, in the heat of the argument they had quite forgotten.

